# Emergency Backwards Whipple for Bleeding: Formidable and Definitive Surgery

**DOI:** 10.1155/2017/2036951

**Published:** 2017-07-03

**Authors:** Cristian Lupascu, Ana Trofin, Mihai Zabara, Alexandra Vornicu, Ramona Cadar, Nutu Vlad, Oana Apopei, Valentin Grigorean, Corina Lupascu-Ursulescu

**Affiliations:** ^1^Department of Surgery, University of Medicine and Pharmacy “Grigore T. Popa”, Str. Universitatii nr. 16, 700115 Iasi, Romania; ^2^Surgical Unit 2, “Sf. Spiridon” Hospital Iasi, Bd. Independentei nr. 1, Iasi, Romania; ^3^Intensive Care Unit, “Sf. Spiridon” Hospital Iasi, Bd. Independentei nr. 1, Iasi, Romania; ^4^Department of Surgery, University of Medicine and Pharmacy “Carol Davila” Bucharest, Bucharest, Romania; ^5^Department of Radiology, University of Medicine and Pharmacy “Grigore T. Popa” Iasi, Str. Universitatii nr. 16, Iasi, Romania

## Abstract

**Introduction:**

During the past decades, the safety of pancreatoduodenectomy has improved, with low mortality and reduced morbidity, particularly in centers with extensive experience. Emergency pancreatoduodenectomy is an uncommon event, for treatment of pancreaticoduodenal trauma, bleeding, or perforation. We herein present a single center experience concerning nontrauma emergency pancreatoduodenectomy for pancreaticoduodenal bleeding.

**Methods:**

From January 2007 to December 2015, from a population of 134 PD (70 males and 64 females, mean age 62.2, range 34–82), 5 patients (3.7%; 2 males and 3 females, mean age 64, range 57–70) underwent one-stage emergency pancreatoduodenectomy for uncontrollable nontrauma pancreaticoduodenal bleeding in our tertiary center.

**Results:**

All the 5 patients underwent a backwards Whipple with a morbidity of 60% and a mortality of 20% (1/5). The other 4 patients were recovered and discharged with a median postoperative length of stay of 17 days (range 14–23).

**Conclusion:**

Emergency pancreatoduodenectomy is a definitive life-saving procedure allowing for a rapid control of bleeding when other less invasive approaches (transcatheter arterial embolization or interventional endoscopy) are exhausted, unavailable, or unsafe. It should be particularly considered in neoplastic disease and tailored by surgeons with a high level of experience in pancreatic surgery.

## 1. Introduction

Pancreatoduodenectomy (PD) is a complex and challenging operation, routinely performed as an elective surgery, mostly to treat pathology of the bile ducts, pancreas, and duodenum. The surgical morbidity and mortality rates of PD have significantly improved during the past decades likely due to increased surgical experience and improved management of complications. In centers with extensive experience, it has become a routine and safe procedure, allowing broadening of indications. On the other hand, emergency pancreatoduodenectomy (EPD), which has been rarely reported as a life-saving procedure for acute pancreaticoduodenal trauma or massive uncontrollable bleeding from ulcers, tumors, diverticula, ruptured aneurysms, or bleeding pseudocysts, is still a very uncommon procedure and an added challenge for the surgeon [1–19]. Given the urgent nature of peripancreatic hemorrhage and complexity of this surgery, the outcome of EPD may be relatively poor [15]. The procedure is often performed under unstable hemodynamic condition and even in shock [1]. The aim of our study is to review and report our institutional experience with EPD in the particular setting of pancreaticoduodenal uncontrollable nontrauma bleeding as there has been no other reported series so far.

## 2. Material and Methods

From January 2007 to December 2015 from a population of 134 PD (70 males and 64 females, mean age 62.2, range: 34–82), 5 patients (3.7%, 2 males and 3 females, mean age 64, range: 57–70) underwent emergency one-stage Whipple procedure for different indications of pancreaticoduodenal massive bleeding. Emergency resection was defined as a PD carried out on the same day of massive bleeding occurrence or deterioration. Intraoperative records were reviewed for operative time, intraoperative blood loss, and transfusion of packed red blood cells. Postoperative records were evaluated for length of postoperative stay and postoperative complications.

### 2.1. Technical Approach

All the patients were operated by the same senior surgeon using a right backwards Whipple procedure. We preferred this approach as far as it offers rapid and early control over the peripancreatic vasculature (superior mesenteric artery (SMA), portal vein, and pancreaticoduodenal arcades), which is the key point in hemorrhagic emergency. Technical details of this modified posterior approach to the SMA and mesopancreas (MP) were widely exposed in one of our previous publications [20]. In brief, the key points are the following:

- Extended Kocher maneuver to expose the pancreas head beyond the aorta, to get full posterior leftwards mobilization of the duodenopancreas.
- The superior mesenteric vein (SMV) is isolated below the pancreas where it passes over the third duodenum and dissected free from the pancreas and uncinate process, with ligation of the right gastroepiploic and inferior pancreaticoduodenal veins. A tunnel is created between the pancreas and the portomesenteric axis towards the hepatic pedicle.
- The retropancreatic dissection is carried on downwards from the inferior border of the Winslow foramen along the Treitz fascia, exposing the left side of the inferior vena cava, the upper margin of the left renal vein and in between; the origin of the SMA is early isolated.
- The plane between the SMV and the SMA is identified as part of the MP.
- Along the adventitial plane of SMA, the MP is dissected and removed “step-by-step,” from the SMA origin until its entrance into the mesentery, using progressive exposure and gentle medial retraction of the portal vein, which is also freed from the MP.
- The superior and inferior pancreaticoduodenal arteries are identified and ligated.
- Complete excision of the connective tissue between the origin of the SMA and the celiac trunk is performed as well.
- We advocate limited dissection along the right side of the SMA in order to avoid extensive removal of the perivascular nervous plexus, resulting in postoperative intestinal motility troubles.
- Already dissected from the SMV, the uncinate process is exposed up to the right side of the SMA. At this stage the posterior wall of the portomesenteric axis is entirely exposed.
- The last step of the resection is the pancreatic neck transection, just in front of the portal vein using a “cold” scalpel.

## 3. Patient 1

A 65-year-old man with significant comorbidities in the past medical history (pulmonary silicosis, myocardial infarction, and recent aortocoronary bypass) presented at the emergency unit for mild dyspnea, moderate to intense epigastric pain, and intermittent melena in the previous 3 weeks. Physical examination revealed a thin, pale, oriented, subfebrile (37.6°) patient. His abdomen was tender in the lower part of the epigastric area, where a deep, irregularly shaped and painful mass was discovered with palpation. Traces of melena were noticed on rectal examination. Laboratory tests showed mild anemia, leukocytosis, and elevated levels of amylase and lipase. The chest X-ray disclosed a small amount of right pleural effusion. Abdominal ultrasound revealed a hydropic gallbladder with dilated common bile duct and a 5 cm hypoechoic structure in the pancreatic head. The main pancreatic duct was enlarged upstream. Multidetector computed tomography (MDCT) disclosed findings consistent with common mesentery and a 5 cm mass in the pancreatic head including a well-defined cystic structure enhancing similarly to the aorta, consistent with the diagnosis of pancreatic pseudocyst (PPC) complicated with pseudoaneurysm (PPA). An enlarged pancreatic duct of 9 mm upstream structure with a suspected communication in between and no overt dilatation of intrahepatic biliary tree were observed. The angio-MDCT related the PPA to the inferior pancreaticoduodenal artery and revealed a replaced right hepatic artery originating from the right-sided superior mesenteric artery (consistent with the common mesentery), with a retroportal course. On the 3rd day postadmission, the patient had a sudden, sharp pain with general pallor, tachycardia, and abdominal tenderness. The systolic arterial pressure dropped to 80 mmHg whilst the hemoglobin and hematocrit levels decreased to 6.2 mg/dl and 17%, respectively. Color Doppler ultrasound noticed a turbulent blood flow inside the PPC, whilst upper digestive endoscopy revealed important transpapillary hemorrhage, confirming the sudden rupture of the PPA with active bleeding inside the PPC. Emergent transcatheter embolization failed to control the bleeding owing to complex arterial feeding. As the patient became hemodynamically unstable, an EPD was performed using a right backwards approach, allowing for steady hemostasis and removal of the PPC. The postoperative course was uneventful, and the patient was discharged 14 days later.

## 4. Patient 2

A 69-year-old woman with medical records of C-virus hepatitis, arterial hypertension, and diabetes mellitus was transferred in emergency from the Gastroenterology department with hematemesis, hematochezia, and decreasing levels of hemoglobin and hematocrit. Physical examination revealed a pale, oriented patient with low arterial pressure of 95/55 mmHg with tenderness in the epigastric area. Upper digestive endoscopy found partially digested blood but no hemorrhagic lesion in the stomach and active massive bleeding from the duodenum. Emergent laparotomy disclosed a bleeding diverticulum of the first jejunal loop, without any other detectable gastric or duodenal hemorrhagic lesion. A segmental enterectomy with end-to-end enteral anastomosis was undertaken. On the 2nd postoperative day, the patient became suddenly hemodynamically unstable, with hypovolemic shock, hematemesis, and dropping of hemoglobin and hematocrit levels. Emergent upper digestive endoscopy found fresh blood in the duodenum with active bleeding at this level, but it failed to identify the source and, thus, to perform any attempt of local hemostasis. Due to the poor hemodynamic status, the patient was straightly brought into the operative room. Intraoperative exploration found out a bleeding in the 3rd duodenum diverticulum with vascular fistula and diffuse duodenal massive bleeding angiodysplasia. EPD was performed as a life-saving procedure. In the immediate postoperative period, the patient developed multiple organ failure and died within the first 24 hours.

## 5. Patient 3

A 70-year-old man with a history of colon cancer (locally advanced mucinous adenocarcinoma of the right colon, requiring right colectomy and adjuvant chemotherapy) presented one year after first surgery with recurrent hematemesis and melena, persistent nausea and vomiting, and weight loss. On general physical examination, he had an anxious look, pallor, tachycardia (96/min), and a large amount of hemorrhagic nasogastric aspirate. Digital per rectal examination confirmed melena. Laboratory tests showed mild anemia. Abdominal ultrasound revealed a hypoechoic area of 55 × 52 × 50 mm tangent to the head of the pancreas. Upper digestive endoscopy identified a 5 cm bleeding exophytic structure located above the inferior flexure of the duodenum, with central ulceration and vascular fistula, highly suggestive of malignancy. Emergency MDCT disclosed a heterogenic mass of 50/52/62 cm on the descendent part of the duodenum with active bleeding, no boundary with the pancreatic head and signs of invasion at this level. Whereas endoscopic procedures consisting in local vasoconstriction drug injection and argon beam coagulation along the tumor mass proved ineffective, urgent surgery was considered as the last and definitive mean of hemostasis. An EPD was performed using the same backwards approach. Pathologic examination concluded duodenal metastases from colon cancer. The postoperative course was complicated by delayed gastric emptying, conservatively treated, and the patient was discharged in good health 16 days after surgery.

## 6. Patient 4

A 57-year-old woman with a past history of virus C hepatitis and chronic liver failure was transferred from another unit and presented with passage of black terry stools, hematochezia, and anemia (7 g/dl hemoglobin). No site of active hemorrhage had been detected either on upper and lower digestive endoscopy or surgical exploration previously undertaken. At admission, the patient was stable, but four hours after, the patient had another episode of massive gastrointestinal bleeding and was transfused with three units of packed red cells. A new upper digestive endoscopy detected partially digested blood with no potentially hemorrhagic lesions in the stomach, large amount of fresh blood in the duodenum but no active bleeding at the time of examination. An emergency angio-CT scan revealed enlarged duodenal varices in the 2nd and 3rd duodenum, tributary to the inferior pancreaticoduodenal veins. As the patient's hemodynamic worsened, surgical hemostasis was decided and multiple bleeding variceal lesions were discovered through a duodenotomy. Repeated attempts at local hemostasis to the bleeding varices by ligatures or stitches proved ineffective so the surgeon decided to perform an EPD with early ligation of enlarged inferior pancreaticoduodenal veins by posterior approach. No complication was noted during the postoperative follow-up other than a persisting bilateral pleural effusion requiring minimal drainage pleurostomy and moderate ascites. The patient was discharged in the 23rd postoperative day.

## 7. Patient 5

A 59-year-old female presented with persistent nausea, vomiting, intermittent hematemesis and melena, and weight loss (10 kg in 2 months). A pale, anxious, and thin patient was observed on clinical examination with BP of 100/70 mmHg and tachycardia of 104/min. The rectal examination confirmed melena. The hemoglobin level was 8.3 mg/dl, and the hematocrit was 31%. Emergency upper gastrointestinal endoscopy revealed fresh blood in the stomach and a fleshy, exophytic, stenotic, and ulcerated tumor arising in the gastric antrum. A biopsy was taken which was reported as a gastric adenocarcinoma. MDCT disclosed nonhomogeneous thickening of the gastric wall up to 21 mm, as well as of the first and second parts of the duodenum. No liver metastases or significantly involved regional lymph nodes were found. The patient was scheduled for radical surgery, but on the 4th day of admission, she presented another episode of massive bleeding and had a transfusion of four units of packed red cells. Whereas the hemorrhage persisted with signs of hypovolemic shock, an emergency gastrectomy was proposed with hemostatic and curative intent. At laparotomy, the gastric tumor was involving the duodenum and the pancreas head. Given the ineffectiveness of an isolated radical gastrectomy, a radical backwards Whipple alongside a subtotal gastrectomy was undertaken. The postoperative course was uneventful, and the patient was able to leave the hospital on the 15th postoperative day.

## 8. Results

The details of patients undergoing EPD, including surgical and postoperative data, are summarized in Table 1.

One patient (20%) died of multiorgan failure in the first postoperative day. Postoperative minor complications occurred in 40% of patients. 80% of patients required additional blood transfusion with a mean 4 U of packed red blood cells. Mean operative time was 270 min (range, 240–320 min). Mean hospital stay was 18 days (range, 14–23).

## 9. Discussion

EPD has been scarcely reported mainly for abdominal trauma, with a mortality rate higher than 30% [1]. There are only few isolated cases or short series (3–6) reported about EPD in nontrauma patients, triggered by duodenal perforation and complications of pancreatitis, such as ruptured aneurysms, bleeding pseudocysts or progressive multiple organ failure in severe necrotizing pancreatitis, and hemorrhagic gastroduodenal and pancreatic lesions, including periampullary tumors, duodenal lymphoma and paraganglioma, and pancreatic metastases [2–19]. A literature review performed via PubMed with the keywords “emergent/emergency” and “pancreaticoduodenectomy/pancreatoduodenectomy” identified 22 reported cases of EPD for nontrauma bleeding lesions (Table 2). Up to date, this is the largest reported series of EPD for nontrauma bleeding lesions.

Upper digestive tract bleeding, including duodenal or pancreatic, can be managed by endoscopic or radiological procedures in most patients. However, endovascular approach is occasionally unavailable or ineffective, unsafe, or useless due to neoplastic origin of bleeding or diffuse pattern of hemorrhage.

Surgery is usually indicated for recurrent or massive uncontrolled bleeding, necessitating transfusion of more than 6 to 8 units of blood [19]. Tumoral bleeding is also an indication of embolization, but locally advanced tumors usually have multiple arterial feeders, from the gastroduodenal artery and the superior mesenteric artery, rendering embolization difficult or insufficient. Moreover, interventional radiological procedures are only palliative for neoplasia and even for late bleeding complications of necrotizing pancreatitis [4]. Therefore, in specific situations, EPD is a definitive therapeutic procedure, especially in patients with bleeding malignancies as it allows either rapid and steady hemostasis or removal of the lesion.

In our series, indication for EPD was based on clinical (2) and intraoperative decision (3).

Reported mortality for EPD in nontraumatic patients is also high, up to 40% [4]. Overall mortality on the 27 reported cases of EPD for nontrauma bleeding is 11.11%. Mortality was 20% in our series, related rather to the patient's poor clinical status than to the procedure. Minor postoperative complications occurred in 2 cases in our series (40%) and were treated conservatively (delayed gastric empting and pleural effusion).

EPD is the option of choice in selected emergent bleeding nontrauma situations, provided that the procedure is performed by a surgeon with high level experience in pancreatic resections. There are some technical difficulties related to the EPD: (1) The special pattern of pancreaticoduodenal arterial and venous arcades. (2) A soft pancreas is a frequent situation in EPD, associated with an increased risk for pancreatic fistula [8]. Hence, we usually perform a stented end-to-side pancreatojejunal anastomosis “duct-to-mucosa” which seems to be more appropriate in these cases. (3) Normal-sized pancreatic and bile ducts are also challenging for pancreatic and biliary anastomosis. No upstream hepatic or pancreatic duct dilatation raises concerns either in hepaticojejunostomy or in pancreatojejunostomy tailoring, leading to an increased postoperative morbidity. (4) The need for urgent vascular access to the arterial feeder renders “artery-first” approaches to the pancreaticoduodenal vasculature more suitable in EPD for bleeding lesions [20]. We used in all our cases a right posterior Whipple procedure, one of the modified “artery first approaches” whereas it affords an early control over the peripancreatic vasculature (SMA, PV, GDA, and inferior pancreaticoduodenal vessels). We think that this approach is associated with less bleeding, so much more that the pancreatic transection is performed the last, just before the removal of the specimen. (5) A radical EPD is required in malignancies.

## 10. Conclusion

Emergency pancreaticoduodenectomy for bleeding may be a definitive life-saving surgical procedure allowing for rapid control of bleeding and securing the hemostasis whether other less invasive approaches (transcatheter embolization or interventional endoscopy) are exhausted, unavailable, or unsafe. Early vascular control by right backwards approach during EPD is a useful tool over the bleeding, decreasing the operative blood loss. The procedure should be particularly considered in malignancies and tailored by surgeons with high level of experience.

## Figures and Tables

**Table 1:** Patients' data.

	Case 1	Case 2	Case 3	Case 4	Case 5	Mean	%
Age	65	69	70	57	59	64	—
Diagnosis	PPC with PPA	Diverticulum angiodysplasia	Duodenal metastases of colon cancer	Duodenal varices	Locally advanced gastric cancer		
Site of bleeding	Pancreatic duct	Duodenum	Duodenum	Duodenum	Stomach		
Nature of lesion	Benign	Benign	Malignant	Benign	Malignant		
Blood loss during EPD	950	1800	800	1200	1150	1180	—
Operative time (min)	250	320	240	260	280	270	—
Postoperative complications	0	Multiple organ failure	Delayed gastric emptying	Pleural effusion ascites	0	—	40%
Postoperative death	0	1	0	0	0	—	20%
Postoperative hospital stay	14	1	16	23	15	18	—

**Table 2:** Nontrauma emergency pancreaticoduodenectomy for bleeding—literature review [6–19].

Paper	Number of cases	Indication	Mortality rate
Eftimie et al. [6]	1	Chronic pancreatitis, intracystic bleeding, and communication with posterior wall of the duodenum	0
Gold and Bordley [7]	4	3 adenocarcinoma of the duodenum/pancreatic head, 1 ulcerated carcinoid tumor of the duodenum	0
Gulla et al. [8]	3	Bleeding pancreatic ductal adenocarcinoma Pancreas and duodenal liposarcoma Bleeding duodenal ulcer	0
Hecker et al. [9]	1	Duodenal GIST	0
Maeda et al. [10]	1	Pancreatic metastasis from renal cell carcinoma in a patient with von Hippel-Lindau disease	0
Parray et al. [11]	1	Duodenal paraganglioma	0
Perera et al. [12]	1	Juxtaampullary duodenal varices due to arteriovenous malformation around the pancreatic head	0
Standop et al. [13]	3	(i) Postinterventional severe necrotizing pancreatitis	33.3%
		(ii) Postsurgical complications after distal gastric resection according to Billroth II	
		(iii) Carcinoma of the papilla of Vater	
Stratigos et al. [14]	1	Duodenal lymphoma	0
Tsai et al. [15]	2	Internal bleeding after partial pancreatectomy; bleeding duodenal lymphoma	0
Tuech et al. [16]	1	Chronic pancreatitis-induced massive bleeding	0
Ujiki et al. [17]	1	Dieulafoy lesion of the duodenum	0
Vladov et al. [18]	1	Pancreatic head mass and bleeding from papilla of Vater after ERCP with papillosphincterotomy	0
Z'graggen et al. [19]	2	Penetrating postpyloric ulcer Ampullary tumor	50%
Present study	5	PPC with PPA Duodenal diverticulum angiodysplasia Duodenal metastases of colon cancer Duodenal varices Locally advanced gastric cancer	20%
